# Logarithmic Fuzzy Entropy Function for Similarity Measurement in Multimodal Medical Images Registration

**DOI:** 10.1155/2020/5487168

**Published:** 2020-02-12

**Authors:** Yu Miao, Jiaying Gao, Ke Zhang, Weili Shi, Yanfang Li, Jiashi Zhao, Zhengang Jiang, Huamin Yang, Fei He, Wei He, Jun Qin, Tao Chen

**Affiliations:** ^1^Changchun University of Science and Technology, School of Computer Science and Technology, WeiXing Road, Changchun 130022, China; ^2^Department of General Surgery, Nanfang Hospital, Southern Medical University, Guangzhou 510515, Guangdong Province, China

## Abstract

Multimodal medical images are useful for observing tissue structure clearly in clinical practice. To integrate multimodal information, multimodal registration is significant. The entropy-based registration applies a structure descriptor set to replace the original multimodal image and compute similarity to express the correlation of images. The accuracy and converging rate of the registration depend on this set. We propose a new method, logarithmic fuzzy entropy function, to compute the descriptor set. It is obvious that the proposed method can increase the upper bound value from log(*r*) to log(*r*) + ∆(*r*) so that a more representative structural descriptor set is formed. The experiment results show that our method has faster converging rate and wider quantified range in multimodal medical images registration.

## 1. Introduction

Multimodal medical images are important for observing tissue structures clearly in clinical practice, such as MRI/T1, MRI/T2, and MRI/PD images. To integrate multimodal information, multimodal registration is important in practical application [1, 2].

It is hard to find relevant information on multimodal medical images because of different weighting properties. To solve this problem, many research works try to find the potential relationship based on intensity value. Whereupon, mutual information (MI) [3] has been extensively applied for multimodal medical image registration. In 2004, Russakoff et al. used MI on medical images registration [4], while it is sensitive on implementation decisions as well as small convergence rate. In 2010, Loeckx et al. used conditional mutual information as a new similarity measure in nonrigid image registration [5]. However, it has an obvious drawback in time consumption. There is an alternative method to decrease the algorithmic complexity, which simulates one modality with the other. This needs a descriptor set to inherit the structure or richness of original modality with the other modality's character expressed. For example, in 2008, Wein et al. [6] registered ultrasound and CT with the simulation of ultrasound images. And In 2013, Xu et al. [7] registered CT image to ultrasound image with simulating the ultrasound image, which has many objective restrictions and the accuracy depends on manual landmark. We are interested in a general structural representation, so these specific approaches are not applicable. The universal adaptability and computational complexity seem incompatible. However, in 2012, Wachinger and Navab [8] proposed the descriptor set based on middle-type artificial modality. It has both general adaptability and low complexity, which is the method we will improve in this article. In the same year, Heinrich et al. computed third-type modality by MIND descriptor set [9]. The descriptor is suitable for different modality-group registration. However, it is affected by rotational variant and cannot recover strong rotations. The descriptor needs ability to express the anatomical feature presented in both modalities. In 2015, Oktay et al. [10] presented a structural representation, which is trained by structured decision forest, namely, Probabilistic Edge Map (PEM). This method lacks a certain generalization ability, which requires manual intervention to adjust parameters and repeated training steps alone. In 2016, Simonovsky et al. [11] applied a deep convolutional neural network (CNN) algorithm to multimodal image registration and optimized it with a continuous framework. The trained network can output the convolutional descriptor set which can address the binary classification between aligned and misaligned, although it causes a huge computing cost in iteration. In 2017, Cao et al. [12] overcame the problem of CT-MRI pelvic image registration by establishing a bidirectional image synthesis. The shortcoming of synthesis methods is the feasibility in other image modalities, which limits their clinical applications. In 2018, Luo et al. computed the descriptor vector based on a novel variogram-based outlier screening method [13]. However, it focuses on space location relationship and loses sight of potential richness. Most recently, in 2019, Bashiri et al. [14] expressed the descriptor set in high dimensional space, studying potential structures of an image through Laplacian eigenmap. Nonlinear dimensionality reduction from manifold space will result in the loss of original potential information. Since the registration of medical images from different modalities is more affected by substantial intensity variations, we prefer the method that is based on pixel intensity distribution.

### 1.1. Motivations and Main Contributions

In clinical application, different modalities have different display emphases. In this case, a universally adaptable approach has significance in multimodal registration. An alternative method is transferring both different modalities into third-type artificial modalities with carrying original potential information. Wachinger and Navab computed third-type modality by entropy [8]. A structure descriptor set was applied to replace the original multimodal image. It has universality and lower computation complexity. However, we found that the above method (entropy function) is only used for quantifying the uncertainty of patches with limited range.

We propose a logarithmic fuzzy entropy function with wider quantified range, which increases the upper bound value from log(*r*) to log(*r*) + ∆(*r*). The experimental results show that our method has faster converging rate and wider quantified range in multimodal medical image registration.

## 2. Structure Descriptor Set

Descriptor set is a medium to express substantial information of original image such as edge, corner, texture, and gradient. In this article, each descriptor is computed by the intensity distribution, which is generated by a local patch. Furthermore, we find that the descriptor contains the structure and richness information, where richness information exists in the form of quantifying its uncertainty, and then the structure descriptor set consists of these descriptors. Such structure descriptor sets can assist many image processing tasks. An accurate structure descriptor set can express the structure and intensity distribution information, reduce the redundant data, and improve the rate of convergence to the extremum value of algorithm. In addition to the above three advantages, we also transform the multimodal image into a third-type modality simultaneously. Finally, under the same modality, we obtain the similarity value by computing the L1 norm of two corresponding structure descriptor sets.

### 2.1. Entropy Image

Wachinger and Navab proposed a structural representation based on the entropy image [8]. The image is divided into many patches, and each patch has its structural descriptor. Structural descriptors are applied to form a completely new image, which are called structural representation. In the new image, every pixel can be calculated as follows:

(1) $$\begin{array}{c} D_{x,l}^{I}=H\left(I|N_{x,l}\right), \end{array}$$

where *H* is the entropy calculation, *I* is image, *N*_*x*,*l*_ is the square neighborhood, which takes *x* position as centre *l* as side length, and *D*_*x*,*l*_^*I*^ is structure descriptor value of *N*_*x*,*l*_. This method quantifies the uncertainty value of the patch with entropy. But the quantification range is only from 0 to log(*r*), which needs to be optimized.

### 2.2. MIND Descriptor Set

Heinrich et al. proposed the MIND method (morphological independent neighborhood descriptor for multimodal registration) [9]. The characteristics of local self-similarity are used to describe structural information. In this descriptor set, each pixel value is calculated as follows:

(2) $$\begin{array}{c} \text{MIND}\left(I,x,r\right)=\frac{1}{n}\exp\left(\frac{-D_{p}\left(I,x,x+r\right)}{V\left(I,x\right)}\right)\text{,}r\in R, \end{array}$$

where *r* is the neighborhood block, *D* is the correlation between the neighborhoods, and *n* is the normalization constant. Each position *x* of image will be replaced by a vector of size |*R*| when the MIND operation is performed.

## 3. The Method of Measurement Function

The method proposed in this article is based on intensity distribution. The essence is to find a function to compute the descriptors. Each descriptor contains the local information of original image, such as intensity richness of local neighborhood. Richness information exists in the form of quantifying the uncertainty value of local neighborhood. Some measurement functions can quantify the uncertainty of set. Buzug et al. adopted strict convex function instead of Shannon entropy [15]. Subsequently, Pluim et al. proposed F information measure instead of the entropy value in mutual information MI calculation [16]. Experiments showed that the registration results of these F information measurements (strict concave function) can imitate mutual information, and some of them have higher precision. These researches prove that there are some measurement functions that have good performance to quantify the uncertainty set, such as entropy function in chapter 3.1 and strict concave function in chapter 3.2.

### 3.1. The Entropy (M1)

The Shannon entropy of a random variable “A” with a possible value “a” is defined as follows:

(3) $$\begin{array}{c} H\left(A\right)=-\underset{i\in a}{\sum }P\left(A=i\right)\times \;\log\;P\left(A=i\right). \end{array}$$

When we calculate the variation of intensity, which occurs in the same position, image gradient is always used for image processing [17]. But, it depends on similarity value and is not suitable for describing the structure detail. A more general concept is to quantify the uncertainty content or, analogously, the bound for a lossless compression, as stated by Shannon's theorem. The entropy function originates from the field of thermodynamics at the earliest. It can measure the uncertainty of variable information. When there are intersections between two images, the correlation of the two images can be calculated with *I*(*A*, *B*)=*H*(*A*)+*H*(*B*) − *H*(*A*, *B*). The above theory is derived from the mutual information MI algorithm [4].

Shannon pointed out that the measurement function of uncertainty should satisfy the following three prior conditions:

1. Continuity condition: *f*(*p*_1_, *p*_2_,…, *p*_*k*_) should be a continuity function of (*p*_1_, *p*_2_,…, *p*_*k*_).
2. Monotonicity: under the equal probability *f*(1/*r*, 1/*r*,…, 1/*r*) = *g*(*r*). *g*(*r*) should be the increasing function of *r*.
3. Additivity condition: when the value of a random variable is obtained from multiple trials rather than one trial, the uncertainty of the random variable in each experiment should be additive.

Condition 1 and 2 mean that the function must have the ability to quantify the uncertainty of the information. Condition 3 is used for multiple information sources. For example, we measure the occurrence probability of each event in set *X* as follows: (*p*_1_, *p*_2_,…, *p*_*n*_). The probability of each event in set Y is as follows: (*q*_1_, *q*_2_,…, *q*_*m*_). We statistic the entropy of the joint information source *X*, and Y is equal to the sum of the entropy of the information sources *X* and *Y*. *H*(*XY*)=*H*(*X*)+*H*(*Y*).

(4) $$\begin{array}{c} H_{nm}\left(p_{1}q_{1},p_{1}q_{2},\ldots ,p_{1}q_{m},p_{2}q_{1},\ldots ,p_{n}q_{m}\right)=H_{n}\left(p_{1},p_{2},\ldots ,p_{n}\right)+H_{m}\left(q_{1},q_{2},\ldots ,q_{m}\right),\\ \overset{n}{\underset{i=1}{\sum }}p_{i}=1,\\ \overset{m}{\underset{j=1}{\sum }}q_{j}=1,\\ \overset{n}{\underset{i=1}{\sum }}\overset{m}{\underset{j=1}{\sum }}p_{i}q_{j}=1. \end{array}$$

The purpose of this article is simply to find a function that can count the uncertainty of a patch (i.e., satisfy conditions 1 and 2). So, it is not necessary to count the joint uncertainty between any patches.

Entropy is not the only function that can describe the uncertainty of information. Wierman studied the uncertainty measure of information entropy under a rough set [18]. Düntsch and Gediga studied the problem based on knowledge granularity measurement [19]. Yumin et al. proposed several uncertainty measures of neighborhood granule, which had good performance in neighborhood systems [20]. Huang and Wen found that the strict concave function can also calculate the uncertainty of the information and discussed the relationship between the entropy and strict concave function [21]. Wei et al. discussed the uncertainty metric based on fuzzy entropy systematically [22]. In this article, we have introduced three other strict concave functions for the coming experiment (see 3.2 for details).

### 3.2. Strict Concave Function

If function *f*(*x*) is defined in the interval *I*, there are two points *x*1 and *x*2 in I. For any *λ*∈(0, 1) it has

(5) $$\begin{array}{c} f\left(\lambda x_{1}+\left(1-\lambda \right)\;x_{2}\right)\;>\;\lambda f\left(x_{1}\right)+\left(1-\lambda \right)f\left(x_{2}\right). \end{array}$$

According to the definition and properties of strict concave functions, we propose three functions:

1. *f*_1_(*x*)=−[*x* log *x*+(1 − *x*)log(1 − *x*)],  *x* ∈ (0,1], assign 0 ×  log 0 ≔0
2. *f*_2_(*x*)=*x∗* exp(1 − *x*)+(1 − *x*)*∗* exp(*x*) − 1, *x* ∈ (0,1]
3. *f*_3_(*x*)=*x*/(1+*x*) − *x*/2,  *x* ∈ (0,1]

**Figure 1:**
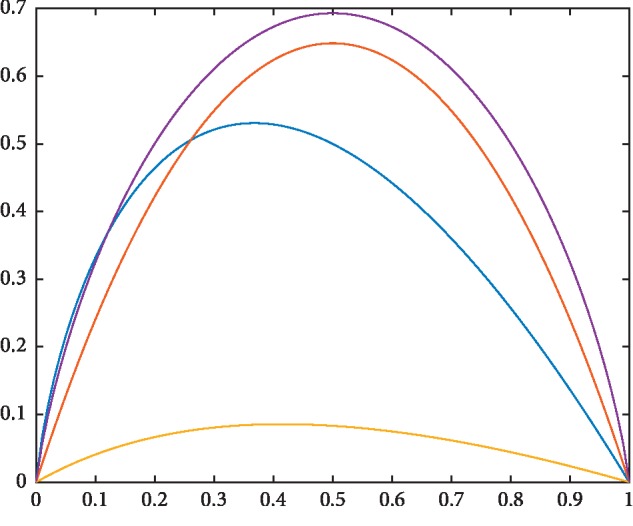
Yellow curve: *y* = *x*/(1 + *x*) − *x*/2; blue curve: *y* = −*x*log(*x*); purple curve: *y* = −[*x*log(*x*) + (1 − *x*)log(1 − *x*)]; orange curve: *y* = *x* *∗* exp(1 − *x*) + (1 − *x*)exp(*x*) − 1.

*f*1(*x*) and *f*2(*x*) are fuzzy entropy in the strictly concave function. *f*3(*x*) is just a strictly concave function rather than a fuzzy entropy function. *f*1 function was presented by De et al. and called logarithmic fuzzy entropy function [23]. *f*2 function was presented by Pal NR et al. and called exponential fuzzy entropy function [24]. The images of four functions are shown in Figure 1.

### 3.3. From Entropy Function to Strict Concave Function

**Theorem 1 (see [25]).**

The intensity value *x*_*i*_*i* ∈ {1,2,3,…, *r*}. According to the definition of entropy function, its range is 0 ≤ *H*(*A*) ≤  log(*r*).For certain *i*=[1, *r*], if *P*(*x*_*i*_)=1, the minimum *H*(*A*)=0. For all *i*=[1, *r*], if *P*(*x*_*i*_)=1/*r*, then maximum *H*(*A*)=log(*r*).

Theorem 1 illustrates that the entropy function can distinguish the dispersion of the probability distribution. For example, a monochrome image contains the least amount of information. And its intensity probability is only distributed at one point, which proves that the set (i.e., image) contains the smallest uncertainty of information. So, the minimum of entropy is 0. We make the hypothesis that there are 256 gray levels (*r* = 256) in the image. Besides, the number of pixels in any gray level is equal, and the gray probability distribution of image satisfies the uniform distribution. At this time, the set (i.e., the image) contains the largest information uncertainty, and the maximum of entropy is log(256).

**Theorem 2 (see [15]).**

If *f*(*x*) is a differentiable strict concave function, then *f*(*x*) has the generalized subadditivity. When ∀ *x*_1_, *x*_2_, *θ* ∈ *R*^+^ and 0 < *θ* ≤ *x*_1_ ≤ *x*_2_, the following inequality is established:

(6) $$\begin{array}{c} f\left(x_{1}-\theta \right)+f\left(x_{2}+\theta \right)<f\left(x_{1}\right)+f\left(x_{2}\right), \end{array}$$

the variable *x* in the function *f*(*x*) represents the probability in medical image registration, so *x* ∈ [0,1], ∑*x*_*i*_=1, *i* ∈ {1,2};  0 ≤ *x*_1_ − *θ* < *x*_1_∩*x*_2_ < *x*_2_+*θ* ≤ 1; formula (6) shows that *f*(*x*_1_)+*f*(*x*_2_) has the maximum value at *x*_1_=*x*_2_=1/2 and minimum value at *x*_1_=0, *x*_2_=1. So, *f*(*x*_1_)+*f*(*x*_2_) can express the measure of the probability distribution. Theorem 3 is obtained when the two sums of the above strictly concave functions are generalized to the sum of the *r* terms.

**Theorem 3 .**

If the function *f*(*x*) has the strict generalized subadditivity, *x*_*i*_(*i*=1,2 …, *n*) indicates the probability of gray value (*i*) in the image, and ∑_*i*=1_^*n*^*x*_*i*_=1. Then uncertainty measurement *M*=∑_*i*=1_^*n*^*f*(*x*_*i*_) can get the maximum value at *x*_*i*_=1/*n*,  (*x*_1_=*x*_2_=*x*_*i*_=⋯=*x*_*n*_) and minimum value at *x*_*i*_=1, *i* ∈ {1,2,3 …, *n*}.

Theorem 2 and Theorem 3 illustrate that the strict concave function can discriminate the probability distribution. When the histogram of the probability distribution is closer to a uniform distribution, the measured value of the strict concave function is the largest; if the distribution is concentrated on an individual point, the measure of the strict concave function is the smallest.

### 3.4. Advantage of Logarithmic Fuzzy Entropy Function

This new function improves the performance by extending the quantification range of patch. Through mathematical derivation, Wachinger and Navab used entropy to quantify a single patch, the upper bound is log(*r*) [8]. However, logarithmic fuzzy entropy function has better symmetry, and it can increase the upper bound from log(*r*) to log(*r*)+Δ(*r*), where *r*=min(*l*^2^, 2^*n*^) is the variety degree in patch; *l* is the side length of patch; *n* is the bit depth of image; Δ(*r*) is monotone increasing function of *r*. In most situation, the magnitude of *l*^2^ and 2^*n*^ is depending on the requirement of performance. No matter in which situation, logarithmic fuzzy entropy function has good performance in quantifying the uncertainty of the patch. Experiments 5.2 and 5.3 show that logarithmic fuzzy entropy function brings faster convergence rate than entropy in multimodal registration, and the convergence rate will increase as *r* increases.

Logarithmic fuzzy entropy function can bring a more representative structure descriptor set. First of all, we need assume that when probability *p*=1 in logarithmic fuzzy entropy function, namely, *M*_2_(1)=0 ×  log 0. This situation means the patch we calculated is a monochrome patch, so we assign 0 ×  log 0 ≔0. The medical image is stored by two bytes per pixel and the bit depth is *n* (*n* ≤ 16), so the variety degree of the patch *r*=min(*l*^2^, 2^*n*^). When probabilities of intensity *p*_1_=*p*_2_=⋯=*p*_*r*_=1/*r*, the uncertainty value of patch can reach the upper bound. We make a comparison among the entropy function (M1), logarithmic fuzzy entropy function (M2), exponential fuzzy entropy function (M3), and strict concave function (M4) in Table 1.

We compare the rate of two functions tending to infinity:

(7) $$\begin{array}{c} \underset{r⟶+\infty }{\lim}\frac{\log\left(r\right)}{\log\left(r\right)+\left(r-1\right)\log\left(r/r-1\right)}=0,\;\left(r=2^{n},n=0,1,2,\ldots \right), \end{array}$$

(8) $$\begin{array}{c} \underset{r⟶+\infty }{\lim}\Delta \left(r\right)=\underset{r⟶+\infty }{\lim}B_{2}\left(r\right)-B_{1}\left(r\right)=\underset{r⟶+\infty }{\lim}\left(r-1\right)\log\left(\frac{r}{r-1}\right)=1,\;\left(\;r=2^{n},n=0,1,2,\ldots \right). \end{array}$$

**Figure 2:**
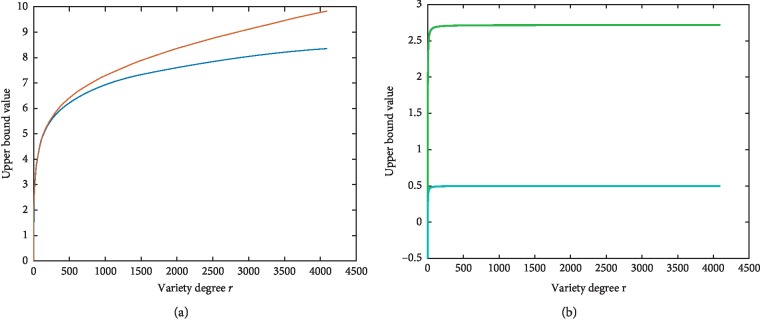
Upper bound function curve. Blue: *B*_1_(*r*); orange: *B*_2_(*r*); green: *B*_3_(*r*); cyan: *B*_4_(*r*), *r* is variety degree.

The curve diagram is showed in Figure 2. There are no much differences between the two function curves when *r* is less than 256. But in medical image, *r* is more than 256. The Δ(*r*) becomes more bigger as variety degree *r* (i.e., *r*=2^*n*^) increases; however, that difference value Δ(*r*) will converge at 1 as shown in formula (8). The larger upper bound brings the wider quantification range, for example, in 256 gray-scale images, the M2 can increase 18% quantification range than M1. Thus, we can compute more representative structure descriptor set under logarithmic fuzzy entropy function (M2).

Theoretically, logarithmic fuzzy entropy function *M*_2_ can compute more representative structure descriptor set because of the larger quantification upper bound. But, the upper bound function *B*_3_ and *B*_4_ converge at 2.705 and 0.496 early. That means before the convergence, *M*_3_ and *M*_4_ can quantify the uncertainty of the image, but when *r* approaches the value of convergence, the upper bound cannot increase as *r* increases.

## 4. Experiment Process

**Figure 3:**
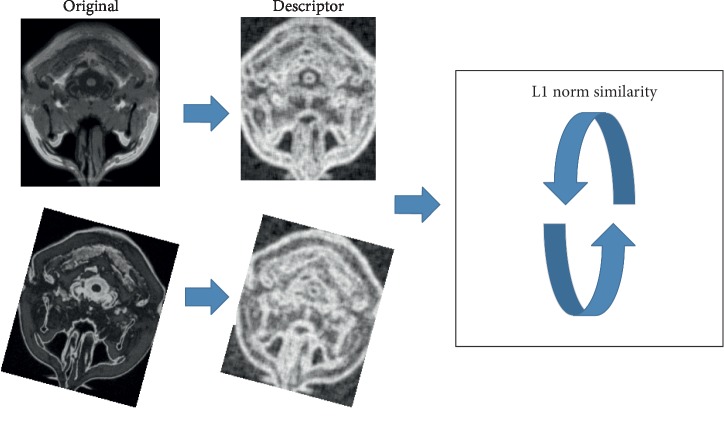
This figure shows the process from the original image to registration. We use L1 norm as similarity measure.

Figure 3 shows the process of the experiment, where we use L1 norm to calculate *S*. The similarity equation can be abstracted as follows:

(9) $$\begin{array}{c} S=\text{MAD}\left(A,T\left(B\right)\right). \end{array}$$

The most similarity status of images *A* and *B* is found by using the spatial transformation *T* and the “MAD” similarity is measured by using the L1 norm. Our target is to find the structure descriptor set *D*^*A*^, *D*^*B*^ to replace *A* and *B*. The similarity equation is converted to

(10) $$\begin{array}{c} S=\text{MAD}\left(D^{A},D^{T\left(B\right)}\right). \end{array}$$

### 4.1. Calculate Descriptor Set

**Figure 4:**
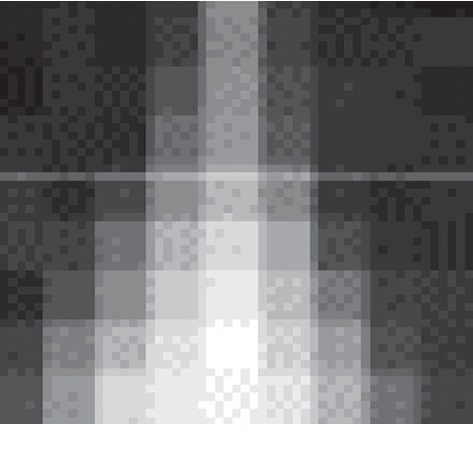
9 × 9 patch *Y*.

A patch *N*_*x*,*l*_ is formed by taking pixel *x* as a centre and *l* as the side length. Taking Figure 4 as an example, patch *Y* has 81 pixels and the side length *l* equals 9. We statistic the intensity histogram and substitute the probability of intensity value into four strict concave functions.

(11) $$\begin{array}{c} M_{1}\left(x\right)=\underset{i}{\sum }-x\;\log\left(x\right),\;x\in \left(0,1\right],\\ M_{2}\left(x\right)=\underset{i}{\sum }-\left[x\;\log\left(x\right)+\left(1-x\right)\log\left(1-x\right)\right],\;x\in \left(0,1\right],\\ M_{3}\left(x\right)=\underset{i}{\sum }\left[xe^{1-x}+\left(1-x\right)e^{x}-1\right],\;x\in \left(0,1\right],\\ M_{4}\left(x\right)=\underset{i}{\sum }\left[\frac{x}{1+x}-\frac{x}{2}\right],\;x\in \left(0,1\right]. \end{array}$$

*M*_1_ is entropy function, *M*_2_ is based on logarithmic fuzzy entropy function, *M*_3_ is based on exponential fuzzy entropy function, and *M*_4_ is based on normal strictly concave function. We replace (1) with the above four functions and get (12).

(12) $$\begin{array}{c} D_{x,l}^{A}=M_{k}\left(\left.A\right|N_{x,l}\right),\;k=1,2,3,4. \end{array}$$

**Figure 5:**
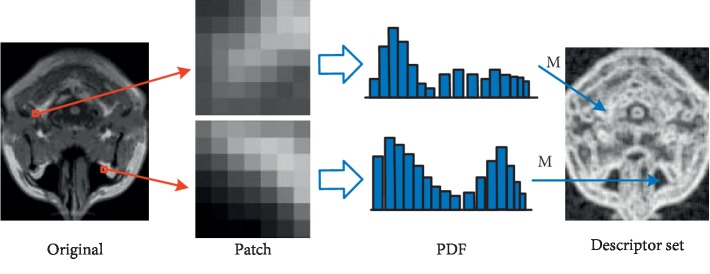
Illustration of the process of computing structure descriptor set. The original image is divided into many patches, and the centre and neighborhood are selected in each patch. The PDF is generated by the statistical histogram of the patch. All the grayscale probabilities of single patch are substituted into the measure function *M* to obtain uncertainty values, namely, descriptor value. Finally, the descriptor value is stored in the corresponding location to create descriptor set [8].

It is available to calculate the uncertainty value of patch *Y* by formula (12). The process from original to descriptor set is shown in Figure 5.

According to the thought of Wachinger and Navab [8], an image is decomposed into several patches, and the respective descriptor values of each patch are calculated by entropy function. In this article, we want to improve the quantification range of descriptor values by the logarithmic fuzzy entropy function and verify the relationship between the quantification range and the speed of convergence. Logarithmic fuzzy entropy function and other strict concave functions have already been discussed in chapters 3.2–3.4.

### 4.2. The Weighting and Patch

**Figure 6:**
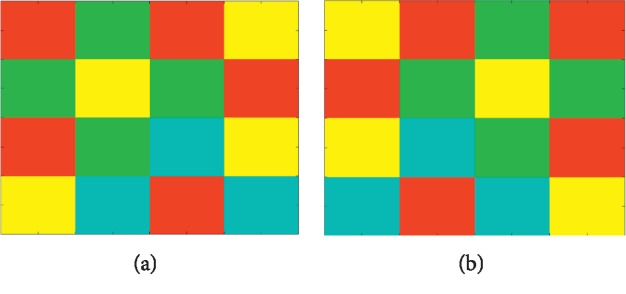
Two patches with symmetrical structure will generate duplicate values because they have the same histogram.

**Figure 7:**
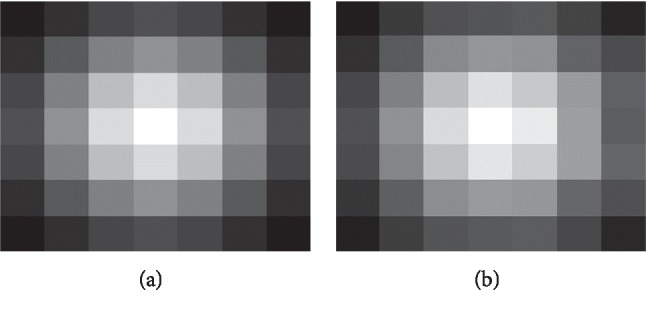
(a) Gaussian weight map; (b) modified weight map.

If two patches have the same intensity value histogram but the structure is different, it will result in the same descriptor value such as in Figure 6. To distinguish that situation, we quote Gaussian weights and modified weighting (Figure 7) from the original author's article [8]. There is a spatial weighting function *ω*  : *N*_*x*,*l*_⟶*ℝ*. Assigning a weight to each patch location, the histogram update changes to

(13) $$\begin{array}{c} \forall y\in N_{x,l}:h_{x}\left[I\left(y\right)\right]⟵h_{x}\left[I\left(y\right)\right]+\;\omega \left(y\right). \end{array}$$

**Figure 8:**
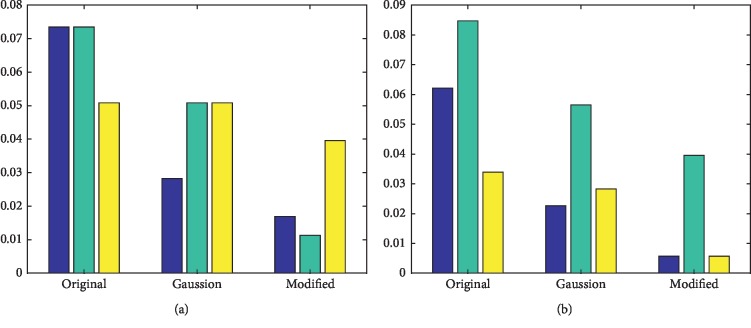
Accuracy error histogram obtained using three weighting methods, blue: T1-T2 data set; green: T1-PD data set; yellow: T2-PD data set, *y* label is accuracy. (a) M1. (b) M2.

Gaussian weighting formula is *ω*(*y*)=*G*_*σ*_(*y* − *c*). The modified Gaussian weighting does not have symmetry compared with the former. In the experiment, these two weights improve the performance of computing descriptor values. It can reflect the local specificity of each point and, at the same time, keep the structure information in the original image. The result is shown in Figure 8.

## 5. Results and Discussion

### 5.1. Experimental Result of Structure Descriptor Set

**Figure 9:**
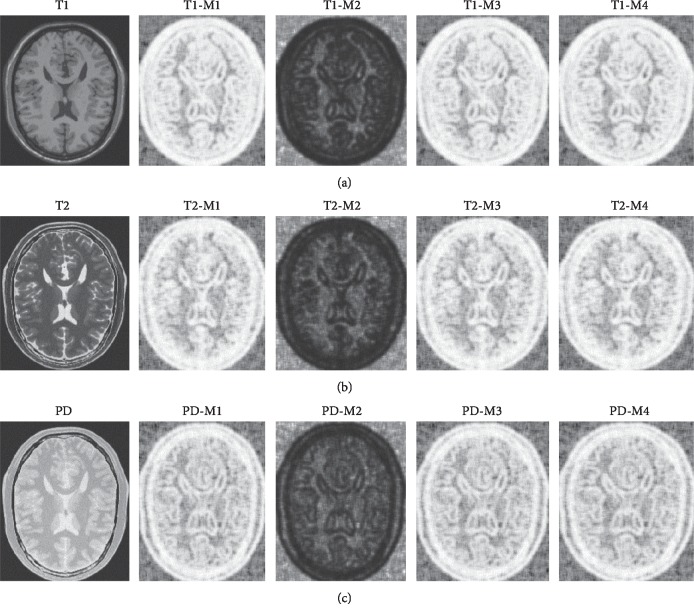
Descriptor set calculated by M1, M2, M3, and M4 under multimodal (T1, T2, and PD).

We use all the descriptor values *D*_*x*,*l*_^*I*^ to replace the *x* position. Structural descriptor sets are shown in Figure 9:

In Figure 9, three different modalities are turned into a third-type artificial modality, and under this modality, we find that they retain the structural information of the original image. The structure descriptor set is computed by four kinds of measurement function. The first row is the result under MRI/T1 modality; second row is the result under MRI/T2 modality; and third row is the result under MRI/PD modality. Each column is the set of structure descriptors calculated under the corresponding measure function. These structure descriptor sets are computed by patch *N*_*x*,*l*_, where *l* is 7.

**Figure 10:**
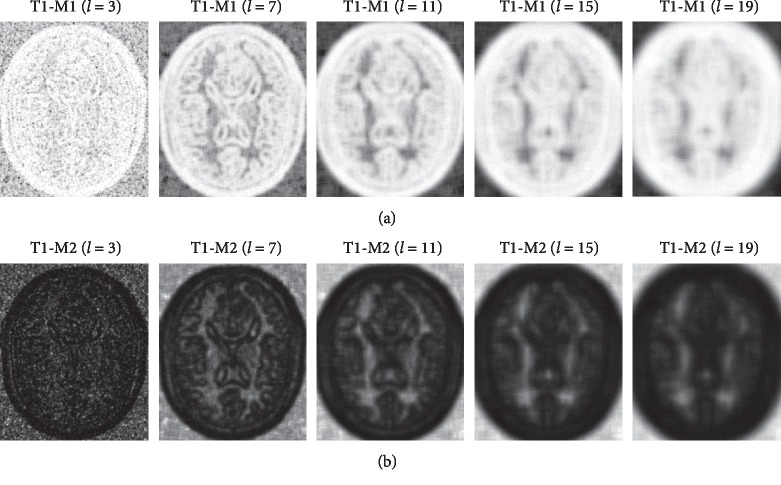
The first row computes T1 modality with M1 function, and the second row computes T1 modality with M2 function. Each column has different patch side length, from left to right *l* = 3, 7, 11, 15, and 19, respectively.

In Figure 10, we alter the side length *l* of the patch, where *l* equals 3, 7, 11, 15, and 19, to calculate the variation of the structure descriptor set. It is found that the image becomes blurred as the *l* increases, which has a similar effect to Gaussian blur. Structurally, the smaller the *l* is, the more sufficient the detail will be. However, statistically, the smaller the *l* is, the duplicate values *D*_*x*,*l*_^*A*^ will get more because the probability distribution of repetition will get more. The bigger the *l* is, the more accurate the value will be because the phenomenon of repeating the probability distribution will be greatly reduced. We inspect pixel value in Figure 10 T1-M1(*l* = 3), there are many duplicate values in it. On the other hand, considering the influence of the local noise, a large patch has a strong ability to suppress that influence.

### 5.2. Anti-Rotation Experiment of Changing the Size of Patch (*l*^2^ < 2^*n*^, *r*=*l*^2^)

**Figure 11:**
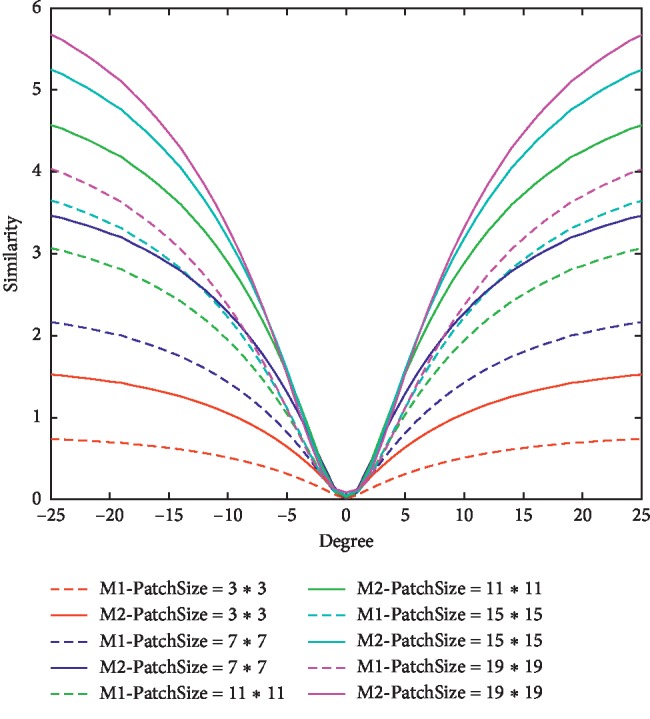
The similarity curves under different patch sizes, red curve *l* = 3, blue curve *l* = 7, green curve *l* = 11, cyan curve *l* = 15, and magenta curve *l* = 19. *l* is the side length of the patch. The dashed curve is M1 function and solid curve is M2 function. *x* label is rotation degree; *y* label is similarity value.

In Figure 11, we verify the relationship between the patch size and convergence rate. We selected the size of patch from 3 *∗* 3 to 19 *∗* 19, and the upper bound will change as patch size changes. In this experiment, we use entropy function (M1) and logarithmic fuzzy entropy function (M2) simultaneously. The dashed and solid curves show that the rate of converging to extremum increases as patch size increases. For each color pair (i.e., in the same patch size), the solid curve is faster than the dashed curve. In this experiment, we keep one image fixed, and the other one rotates along the centre from -25 to 25 degrees. At each angle, the similarity of the two images is measured by M1 and M2. We obtain these data sets from DICOM Library (https://www.dicomlibrary.com). In this data set, there are two different MRI modalities. The image size is 512 *∗* 512 and stored by 13 effective bit depths. There are 47 layers in each modality, so each curve is an average result of 47 layers in two different modalities.

When *l*^2^ < 2^n^, according to Table 1, the upper bound of M1 and M2 are *B*_1_(*l*^2^) < *B*_2_(*l*^2^), where each upper bound has a monotonically increasing relationship with patch size. This experiment proves that the M2 function has faster convergence rate than M1 in the small patch. It can satisfy the requirement of decreasing code running time with the small patch.

### 5.3. Anti-Rotation Experiment of Compressing the Effective Bit Depth (**l**^2^ > 2^**n**^,  **r**=2^**n**^ )

**Figure 12:**
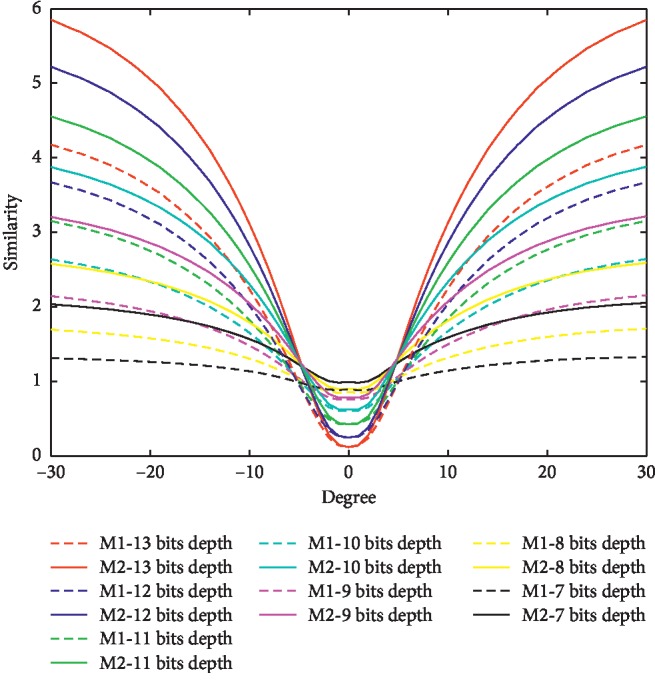
The similarity curves under the condition of different bit depths. The dashed curve is M1 function and solid curve is M2 function; *x* label is rotation degree; *y* label is similarity value.

In Figure 12, we verify the performance of M2 function when the intensity bit depth *n* decrease from 13 to 7. This time, we select the patch size as 65 *∗* 65, because it can contain richer variety. In such a large patch size, the upper bound will change as the bit depth changes. According to Figure 2, the difference of the upper bound of two functions will increase as the variety degree increases. That means, the M2 function's result is better than the M1 function's result in a lager bit depth. There are two different MRI modalities. Each modality has 47 layer images, and each layer is stored in 512 *∗* 512, two bytes, 13 effective bits (i.e., bit depth *n* is 13). So, we make an experiment about decreasing the bit depth *n* from 13 down to 7. They are equal when compressing the intensities down to 1/64, 1/32, 1/16, 1/8, 1/4, and 1/2 of the original image.

We consider one pair color as one group experiment, which contains one dashed curve (M1 function) and one solid curve(M2 function). The different color means different bit depths. For example, the red pair is the original image, the blue pair is using 12 effective bits; the green pair is using 11 effective bits; the cyan pair is using 10 effective bits; the magenta pair is using 9 effective bits; the yellow pair is using 8 effective bits, and the black pair is using 7 effective bits to express the image. Each curve is the average result of 47 couple, and each couple images contain two different modalities. We compute the similarity when rotating one modality image along the centre of the other modality image from −30 degree to 30 degree.

Figure 12 shows, as the bit depth decreases (from 13 to  7), the rate of converging to extremum is going to decrease. No matter what bit depth is, the M2 function can bring a faster converging rate than the M1 function when quantifying the uncertainty of the patch. There are some differences in minimum part when comparing Figure 12 with Figure 11. The minimum increases as the bit depth decreases, which causes the standard deviation of M2 curve to be larger than M1 curve, especially when the bit depth is large. The red pair and black pair curves prove that M2 function can quantify the value of uncertainty in a wider range, which can bring a more representative structure descriptor set. This structure descriptor set is a key point in fast convergence.

### 5.4. Modality-Group Similarity Experiment on Rigid Deformation

The purpose of this experiment is to verify the sensitivity of the algorithm. As slice spacing decreases, it is hard to distinguish adjacent slices, which results in the deviation of many multimodal similarity algorithms. To verify our method's validation, we performed modality-group similarity experiment with 4 different methods: (1) the proposed method in [8] using entropy (M1 function) images, (2) the method using Laplacian method in manifold learning [14], (3) multimodal registration with mutual information (MI) [4], and (4) traditional method with mean absolute differences (MAD). The above result of the experiment is illustrated on Tables 2–4.

**Figure 13:**
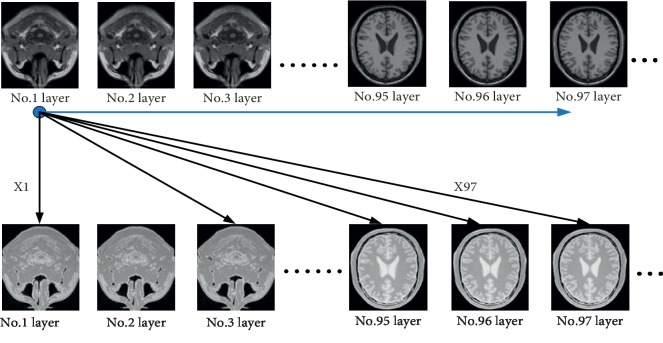
Experiment on accuracy verification.

Finally, we evaluate the performance relationship between these four functions under the condition of side length *l* = 15, Parzen-window estimation, and modified weighting. This data set is from http://www.bic.mni.mcgill.ca/brainweb/. It includes three modalities: T1, T2, and PD. The brain MR image we selected on BrainWeb contains 3% noise and 20% intensity nonuniformity. There are 177 images in each of the three modalities, and we search an image in one of the modalities and then traverse all the images in the remaining modality. We make a comparison by group experiments to reflect the superiority of M1, M2, M3, and M4. All data sets provide standard alignment. Each data set makes 177 times registrations under each function. The experiment process is shown in Figure 13.

**Figure 14:**
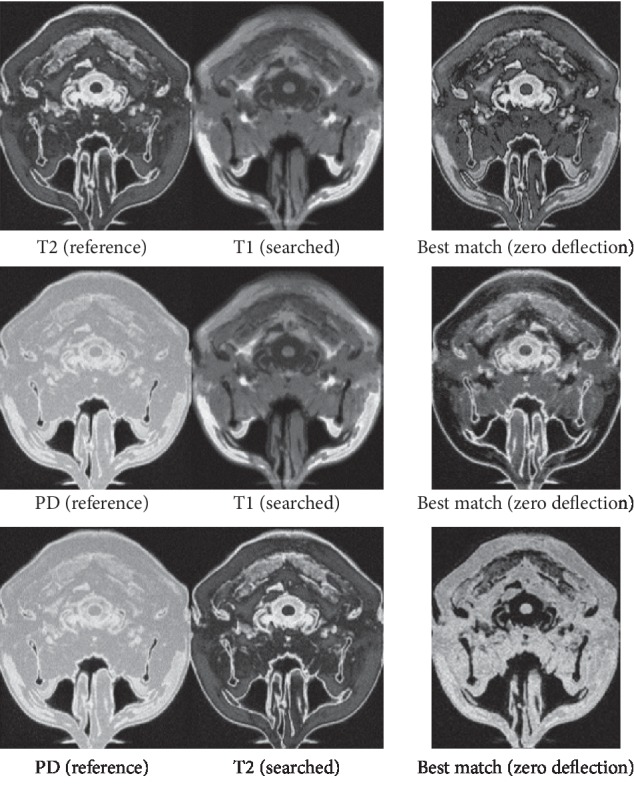
Optimal registration status under each data set.

The blue point moves from left to right, and each action we calculate 177*x* values (i.e. similarity values). Finding the minimum value to judge that if the extreme value position (*P*_*x*_ext__^search^) is corresponding to the given original image position (*P*_*x*_ext__^reference^) or not. The ground truth of each data set is available on downloading the data set. It can be our reference standard state to compare with our experiment results. And we divide the results of comparison into 3 levels within the permissible margin of the error. If the position distance fulfils *P*_*x*_ext__^search^ − *P*_*x*_ext__^reference^=+1, it is called the right deflection; if *P*_*x*_ext__^search^ − *P*_*x*_ext__^reference^=−1, it is called the left deflection; if *P*_*x*_ext__^search^ − *P*_*x*_ext__^reference^=0 , it is called the zero deflection (best match) in Figure 14. That means, the extreme value location should be the same or close as another modal location. Take the PD modality no. 3 layer as an example, we find the most similar image with PD modal from the T1 modal. If the result belongs to any one of no. 2, 3, and 4 layers, we consider these results are in the reasonable error range. And if 2 − 3 = −1, it deflects one layer toward the superior; 3 − 3 = 0, it does not deflect to any layers; the last 3 − 2 = 1, it deflects one layer toward the inferior. If |*P*_*x*_ext__^search^ − *P*_*x*_ext__^reference^| > 1, it means the registration is failed. So, the results are shown in Tables 2–4. (R-right; L-left; D-deflection; N-number; P-probability; Z-zero. For example, LDN is an abbreviation for “left deflection number” SUM = RDN + LDN + ZDN). We make 177 times experiments by each method.

According to the result in Tables 2–4, ZDP has more strict restriction than SUM probability. For M2, it can reach 92.66% in ZDP part, whereas M1 can only reach 84.16%. For MI, it has a slight trend in deflection, which makes LDN and RDN reaching 15 and 12, respectively. For manifold learning, it has a similar result with MI in LDN and RDN. For MAD, it is the worst method in modality-group experiments. The ZNP and SUM probability in MAD only reach 2.26% and 24.86%, respectively.

In contrast to the M2 method, it can be seen that the method has less number in RDN and LDN, which means has stronger ability to distinguish the adjacent slices. The result proves that the MAD method is unsuitable to compute the L2 norm of original multimodal image pairs, especially in M1-M2 group.

### 5.5. Modality-Group Similarity Experiment on Nonrigid Deformation

On the Brainweb databases, we deform one image in each pair with a deformation d_g regarded as the ground truth. Then, we estimate deformation d_c by registering the deformed image and another remained image with different modality one. We calculate the average Euclidean difference of the deformation fields *τ*=1/|*Ω*|∑_*x*∈*Ω*_‖*d*_*c*_(*x*) − *d*_*g*_(*x*)‖ for computing the residual error of the registration.

In Table 5, the configuration for M2 method for deformable registration is: 25 *∗* 25 patches, 16 bins, modified Gaussion weighting, local normalization, Parzen-window estimation and logarithmic fuzzy entropy core function. It can be seen that M2 has the lowest errors in 3 group registration. The results for the M1 (entropy) images are comparable, while the MAD does not perform well.

**Figure 15:**
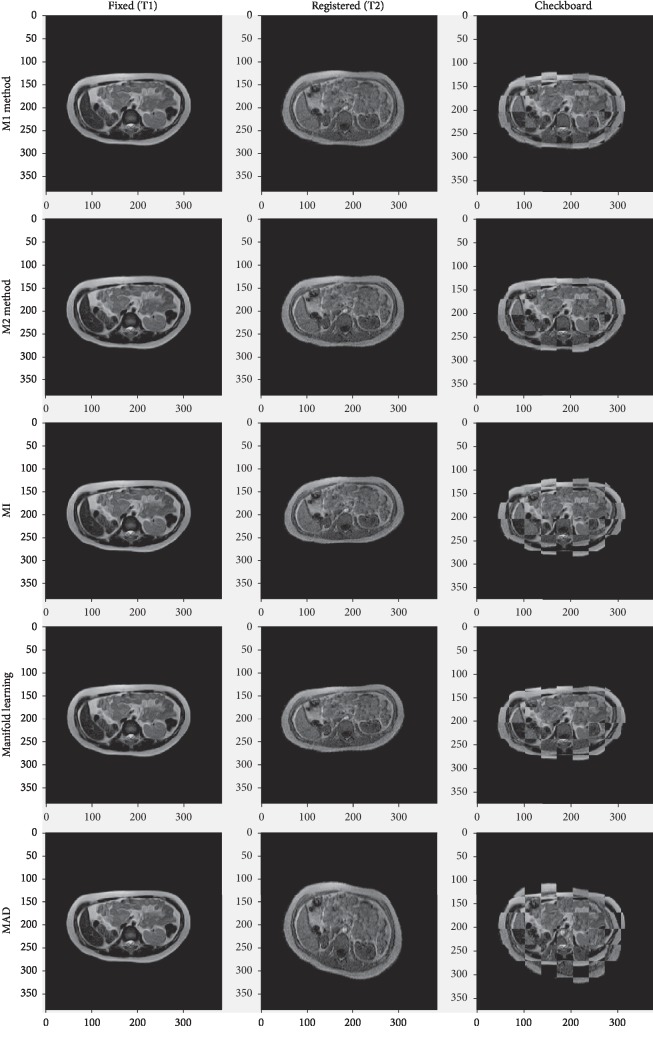
The experiment about one pairwise multimodal (T1-T2) image registration based on abdominal images.

To test the effect of our method in nonrigid deformation, we used abdominal image of MRI-T1 and MRI-T2. The size of image pair is 384 *∗* 384 and a pixel is stored as 12 bits. The result is shown in Figure 15. In each method, we use a common slice (T1 modality) as fixed image, and the other corresponding slice is deformed by 200 manually warping operations such as TPS or affine. In these many fixed deformations, we use 5 methods (M1, M2, MI, manifold ling, and MAD) to find the most similar deformed image of their own. Their most similar result is shown in the Registered (T2) row of Figure 15. We can see that the M2 method has better performance on the image fusion from checkboard.

### 5.6. Translation Experiment

**Figure 16:**
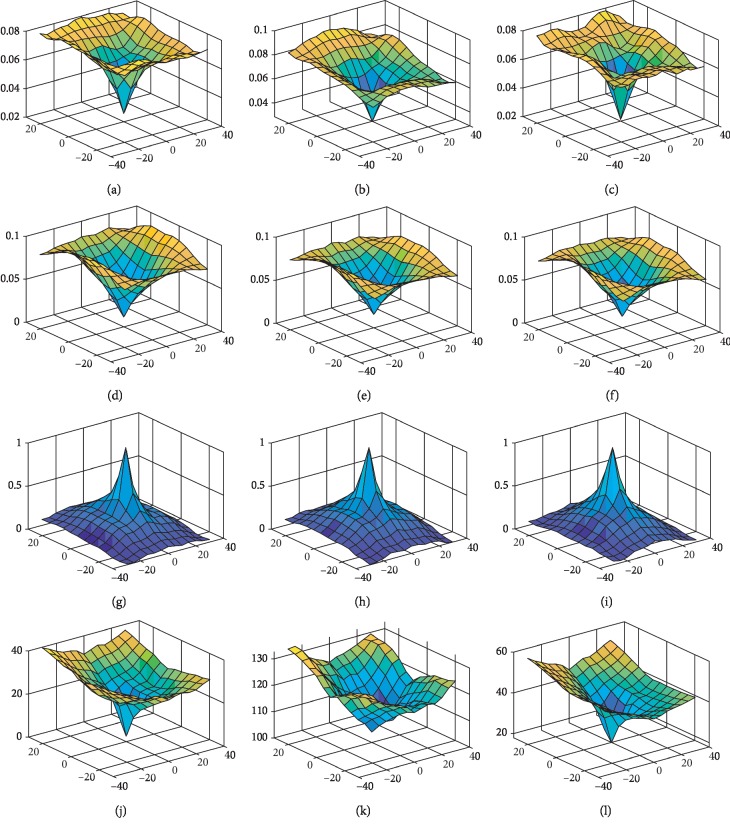
Plot of similarity measures for translation in the *x* and *y* directions. (a) M1, T1-T2. (b) M1, T1-PD. (c) M1, T2-PD. (d) M2, T1-T2. (e) M2, T1-PD. (f) M2, T2-PD. (g) MI, T1-T2. (h) MI, T1-PD. (i) MI, T2-PD. (j) MAD, T1-T2 (k) MAD, T1-PD. (l) MAD, T2-PD.

For the next translation experiment, we compared the performance of M2 (logarithmic fuzzy entropy function) with M1 (entropy function), MI (mutual information), and MAD (L1 norm). The results of the translational experiments under four methods can be seen in Figure 16.

As two images are translated along the *x* and *y* axes in [−40, 40] degrees, the similarity values are calculated by four methods for each degree. For M1 M2 and MAD, as the result is closer to 0, we obtained a stronger correlation between the two images. For MI, as the result is closer to 1, we obtained a stronger correlation between the two images. It can be seen from the smoothness of a curve that M1 M2 and MI are superior to MAD at stability. MI shows a very sharp peak when the translation difference is in the interval [−20, 20], and the system is relatively sensitive. But in [−40, −20] ∪ [20, 40], the method MI is not in our expectations because the similarity between the two images cannot distinguish clearly.

### 5.7. Running Time

Finally, we test the average time of 100 experiments during the normal registration. We select Parzen-windows estimation, modified weighting, and 11 × 11 patch size at the experiment. Running time table is shown in Table 6:

We use MatlabR2016(b) to run code in a normal configuration environment (the process is from the descriptor set to the L1 norm registration). From Table 6, we can see the time of M1-M4 are shorter than MAD, which proves that using structure descriptor sets to calculate the L1 norm similarity is more efficient than using the original image directly. Besides, the M2 function has the shortest running time.

## 6. Discussion

Our proposed logarithmic fuzzy entropy function has a certain contribution on “transform multimodal into third modality.” In this process, the ability of quantified patch is especially important. In Figure 2, we can see that the upper bound of our function is greater than the original function, especially in the large intensity level such as medical images, which can bring us a wide range for quantification. During the rigid and nonrigid registration experiments, the proposed method has good performance in measuring the similarity with an outstanding sensitivity. Regarding 3D, it is inevitable that the computational cost will increase as the dimension increases from 2D to 3D; however, it is not what our method worried about because it is not a complicate job for estimating the PDF (probability density function) of 3D patches. However, in this article, our method is to express the richness of the 2D patch with quantifying the uncertainty by a 1D number. From that view, our method will lose the location information, so we make it up by modified Gaussion weighting in chapter 4.2. If we apply this method on 3D situation, the quantifying process will plunge from 3D to 1D. Besides, there is no suitable 3D weighing that can offset the location information. So, this method does not have robustness in 3D multimodal image registration.

## 7. Conclusion

This article focuses on using the structure descriptor sets (third-type artificial modality) to perform the L1 norm in multimodal registration. We propose logarithmic fuzzy entropy function in the computing structure descriptor set. Through the mathematical derivation and experimental result, this function is more suitable than entropy in multimodal registration. We also tried out other two strict concave functions such as M3 and M4, but they performed worse because of their upper bound curve.

When we quantify the value of a patch by its intensity distribution, the advantages of logarithmic fuzzy entropy function are as follows:

1. Mathematically, it can bring a larger quantification range.
2. Experimentally, it can bring a faster convergence rate in similarity curve.

According to the experiments in chapter 5.4 to 5.6, our proposed method is an effective evaluating approach in similarity of multimodal medical images. It has the following advantages:

1. Inferior computational complexity, which is the process from core function to L2 norm.
2. Universal adaptability, which can work on any modality pair.
3. Higher accuracy, which has strong ability to distinguish similar slices.

This algorithm has an obvious effect when the medical images are stored by high effective bit depth. Because the upper bound of quantification range is monotone, the function of variety degree *r* increases. To avoid duplicated values of different patches which have the same intensity distribution, the patch size will be as large as possible. However, the patch size influences not only the converging rate of similarity value but also the running time; a large patch can increase the running time. Ideally, we want *l*^2^ and 2^*n*^ to be equal. But in practice, patch size depends on many factors such as, original image size, effective bit depth, noise, and requirement of running time. Whatever size it is, the logarithmic fuzzy entropy function is a good choice in the “transfer of multimodal into third-type modality” medical image registration.

## Figures and Tables

**Table 1:** Upper bound table for 4 strict concave functions.

Function	Upper bound	*B*_(*r*⟶*∞*)_
*M*_1_(*p*_*i*_)=∑_*r*_*p*_*i*_log *p*_*i*_	*B*_1_(*r*)=log(*r*)	+*∞*
*M*_2_(*p*_*i*_)=∑_*r*_−[*p*_*i*_log *p*_*i*_+(1 − *p*_*i*_)log(1 − *p*_*i*_)]	*B*_2_(*r*)=log(*r*)+(*r* − 1)log*r*/*r* − 1	+*∞*
*M*_3_(*p*_*i*_)=∑_*r*_[*p*_*i*_exp(1 − *p*_*i*_)+(1 − *p*_*i*_)exp(*p*_*i*_) − 1]	*B*_3_(*r*)=exp(*r* − 1/*r*)+(*r* − 1)exp(1/*r*) − *r*	2.705
*M*_4_(*p*_*i*_)=∑_*r*_[*p*_*i*_/1+*p*_*i*_ − *p*_*i*_/2]	*B*_4_(*r*)=*r*/*r*+1 − 1/2	0.496

**Table 2:** In the T1-T2 data set, the accuracy is within 5 methods.

Method	RDN	LDN	ZDN	SUM	ZDP (%)	SUM probability (%)
M1	7	15	149	171	84.18	96.61
M2	2	8	164	174	**92.66**	98.31
MI	15	12	121	148	68.36	83.26
Manifold learning	10	11	139	160	79.66	90.40
MAD	22	18	4	44	2.26	24.86

**Table 3:** In the T1-PD data set, the accuracy is within 5 methods.

Method	RDN	LDN	ZDN	SUM	ZDP (%)	SUM probability (%)
M1	9	21	140	170	79.10	96.05
M2	3	6	166	175	**93.79**	98.87
MI	29	24	110	163	62.15	92.09
Manifold learning	21	18	125	164	70.62	92.66
MAD	40	72	18	130	10.17	73.45

**Table 4:** In the T2-PD data set, the accuracy is within 5 methods.

Method	RDN	LDN	ZDN	SUM	ZDP (%)	SUM probability (%)
M1	13	20	136	169	76.84	95.48
M2	3	3	169	175	**95.48**	98.87
MI	35	29	99	163	55.93	92.09
Manifold learning	29	29	108	166	61.02	93.78
MAD	42	71	15	128	8.47	72.32

**Table 5:** Registration errors *τ* in mm for various configurations for M2 method.

Sim	T1-T2	T1-PD	T2-PD
M1	0.52	0.61	0.58
M2	0.39	0.43	0.38
MI	0.68	0.76	0.70
Manifold learning	0.71	0.75	0.69
MAD	0.99	2.01	1.25

**Table 6:** Running time table.

Method	Use normal registration framework's time(s)
M1	0.0048
M2	0.0039
MI	0.0070
Manifold learning	0.0096
MAD	0.0672

## Data Availability

Data link available: Brain Web (http://www.bic.mni.mcgill.ca/brainweb/) DICOM Library (https://www.dicomlibrary.com).
